# Territorial song frequency does not signal body size in a song-learning passerine

**DOI:** 10.1038/s41598-025-11589-4

**Published:** 2025-07-16

**Authors:** Lia Zampa, Paweł Szymański, Katarzyna Łosak, Tomasz S. Osiejuk

**Affiliations:** Department of Behavioural Ecology, Institute of Environmental Biology, Faculty of Biology, Adam Mickiewicz University, Uniwersytetu Poznańskiego 6, Poznań, 61-614 Poland

**Keywords:** Body size, Bird song, Sound frequency, Song sharing, Sexual selection, Behavioural ecology, Animal behaviour

## Abstract

The negative relationship between song frequency and body size in birds is well-established across species. However, whether song frequency reliably signals body size and individual quality requires more detailed within-species comparisons, particularly in species with diverse song repertoires. We investigated this relationship in the Ortolan Bunting (*Emberiza hortulana*), a song-learning species with a limited repertoire. Males of this species produce songs composed of two phrases: an initial phrase, which varies across individuals and defines their repertoire, and a final phrase, which is shared across the population. We analysed multiple frequency parameters across both components, accounting for phrase-sharing patterns among males, to assess whether song frequency reflects body size, using tarsus length as a proxy. No significant associations were found between body size and any frequency measure. These findings contribute to the ongoing debate over whether sound frequency signals body size, and by extension, competitive ability, during territorial interactions. Our results highlight the importance of within-species approaches and underscore the need to consider vocal learning dynamics, such as song sharing patterns, when exploring this relationship in song-learning species.

**Supplementary Information:**

The online version contains supplementary material available at 10.1038/s41598-025-11589-4.

## Introduction

The dynamic interplay between biological constraints and the evolution of adaptation has long fascinated biologists, offering an insight into the complexity of life^1^. This interplay becomes particularly challenging when considering how morphological limitations shape the evolution of communication systems. Animal vocalisations serve as signals through which senders convey information to receivers, often influencing their behaviour^2^. In birds, male songs are fundamental to mate attraction and territory defence, making them key targets of sexual selection^3,4^. To function as reliable indicators of individual quality, sexually selected signals are expected to be costly or constrained, ensuring that only individuals of sufficient quality can afford to produce or maintain such signals^5,6^.

The costs incurred in song production can vary across species in different ways^7^. Evidence suggests that performance-related traits, such as the amount of time spent singing^8^specific song timing, like dawn chorus^9^song rate^10,11^and amplitude^12^often serve as indicators of male quality because of the energetic cost they entail. In contrast, the signalling role of sound frequency remains ambiguous^13^. Although it is often described as an index signal, a type of trait that is inherently limited by physical constraints and thus reliably associated with morphology^14^its role as a consistent marker of quality remains debated. Specifically, larger vocal organs and tracts allow for the production of longer sound waves, setting the lowest frequency an animal can efficiently produce^15^. As a result, sound frequency can serve as an honest signal of body size, and potentially of quality, especially when visual assessment is limited. By evaluating a rival’s size from a distance through listening to its songs, individuals can minimise the risk of engaging in costly conflicts, thereby improving territorial defence efficiency^16^.

While the expectation that larger bird species produce lower-frequency songs is widely supported^17–20^this relationship has been poorly explored at the within-species level^13^. Among studies focused on passerine species, some have identified a negative relationship between song frequency and body size^13,21–23^with lower-frequency songs often associated with greater competitive ability and more successful mating^24–26^. However, other studies have found no evidence of size-pitch allometry^13,27^or have reported the opposite pattern, where maximum song frequency correlates positively with body size^28^. Interestingly, songs with relatively higher frequencies, compared to alternative song types within the same species, have been shown in some species to play a significant role in female preferences and competitive interactions^29–32^and have been positively associated with reproductive success^33^. This may be because producing high-frequency sounds may require greater neuromuscular control and increased respiratory effort, which could act as a performance-related signal of individual quality^34^. In this context, not only structural size but also body condition, reflecting an individual’s energetic state, may influence the ability to sustain demanding vocal performance, such as high frequency sound^5,7^.

To determine whether sound frequency signals an individual’s body size, and if body size enforces its honesty, it is essential to conduct within-species comparisons and gather data from a broad range of bird species, accounting for phylogenetic diversity and varying communication strategies. The unclear relationship between sound frequency and body size remains unclear within species can be attributed to several factors. The limited variation in body size among individuals, especially in small passerines, may lack sufficient contrast to reveal significant differences^27^. Additionally, songbirds often exhibit a diverse repertoire of songs, which increases individual variation and complicates the selection of a consistent signal for analysis^28,35^. Different song types, or even different elements within the same song type, may serve distinct functions, such as territorial defence or mate attraction^36–38^. The lack of knowledge of context-dependent signals in the target species can lead to misleading predictions; for example, body size may be more important in territorial aggression than in other behavioural contexts^39^. Finally, in song-learning species, the relationship between body size and sound frequency can be further obscured by other sources of variation related to the learning process^40^. Factors such as the availability of tutors during the early stages^41,42^the benefits of song sharing^43^dialect matching^44^cultural learning and individual recognition^45^ may also influence the sound frequency, potentially masking the effects of body size. This complex scenario highlights the need for more targeted studies on the relationship between song and body characteristics in species with diverse song repertoires, where individuals produce multiple, socially acquired song types.

This study aimed to investigate the relation between body size and acoustic frequency parameters in the song of Ortolan Bunting (*Emberiza hortulana*), a songbird species with a small song repertoire. The Ortolan Bunting seems a good model species to test this relationship for its repertoire and distinctive sharing patterns of song components. Typically, males have two or three song types in their repertoire. Each song type consists of two phrases: the initial phrase (IP), which is shared among some, but not all males, and defines the individual’s repertoire; and the final phrase (FP), which is shared among all males within the population^46^. The sound frequency of song types within a male’s repertoire is stable both within and between seasons, suggesting these frequencies are fixed by biological or learned processes^45^. We hypothesised that if frequency is a reliable indicator of male body size, then larger males would produce lower-frequency versions of their song regardless of the repertoire’s content and size. Specifically, we would expect (a) a general negative correlation between average sound frequency and body size, (b) a negative correlation within shared song types between sound frequency and body size across males, and (c) consistency of this pattern in both parts of the songs, assuming they do not serve distinct communicative functions. In this case, the final phrase, shared by all local males, should allow for direct comparisons among individuals, while the initial phrase, when shared, should exhibit lower frequencies in larger males.

## Methods

### Study species

The Ortolan Bunting is a small migratory passerine species that breeds in southern and eastern Europe and western and Central Asia, and winters in sub-Saharan Africa^47^. This species is commonly found in farmland and open landscapes with scattered trees and sparse shrubs. The population is experiencing rapid declines throughout Europe, largely attributed to habitat loss, agricultural intensification, and illegal hunting^48^.

The fieldwork was conducted in four localities across the Wielkopolska region in western Poland (centres of study plots: N52.235758, E16.648771; N51.851820, E16.739826; N51.819430, E17.088221; N51.543904, E17.692026). In each study area, males usually arrive at the turn of April and May and establish territories along the edges of forests or tree lines surrounded by farmland.

**Fig. 1:**
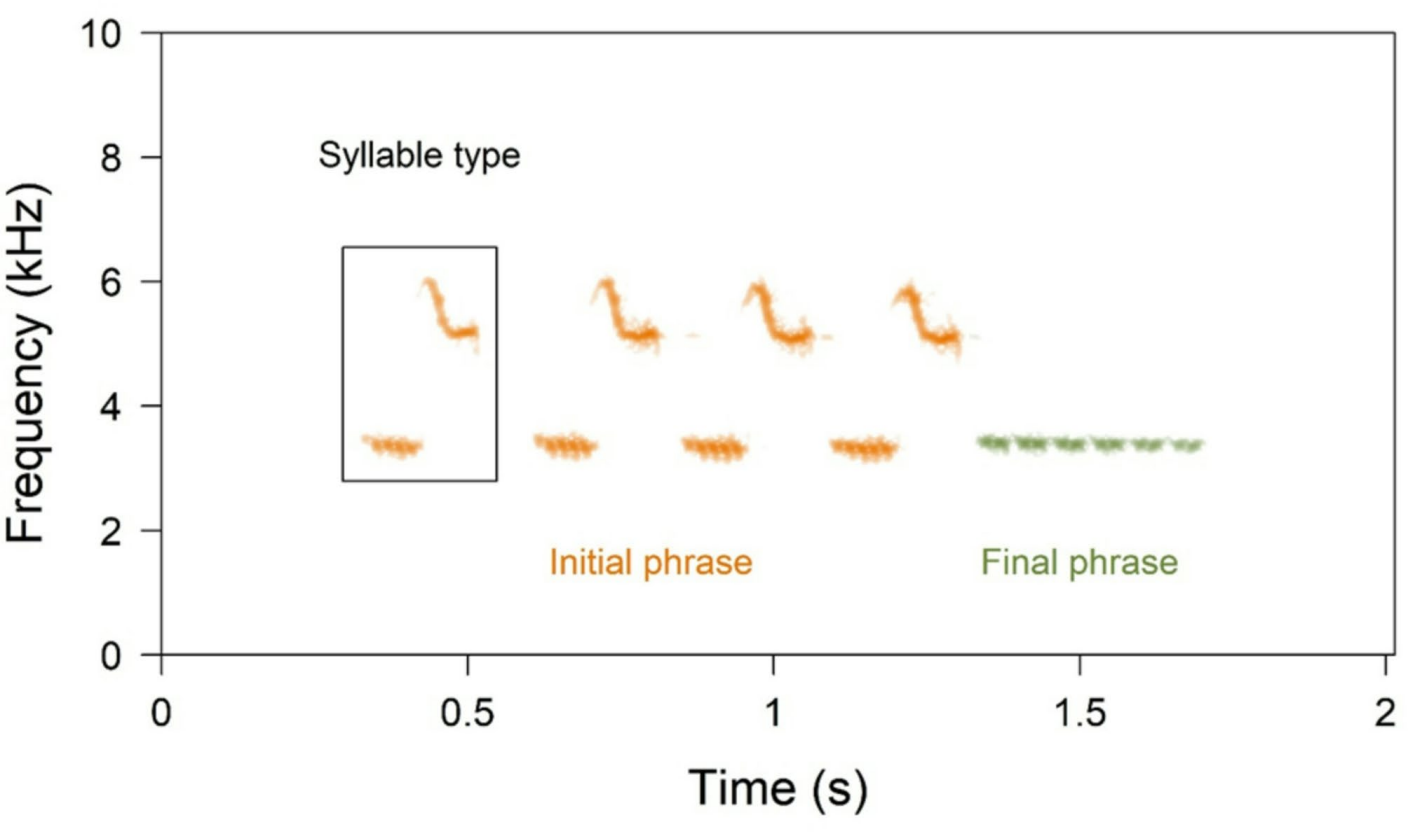
Spectrogram of a typical song from the studied population of the Ortolan Bunting *Emberiza hortulana*. The song components, named **initial phrase (IP)** and **final phrase (FP)** are highlighted with colors. The distinctive shape of the **syllable types** defines the specific song type within the individual repertoire.

**Fig. 2:**
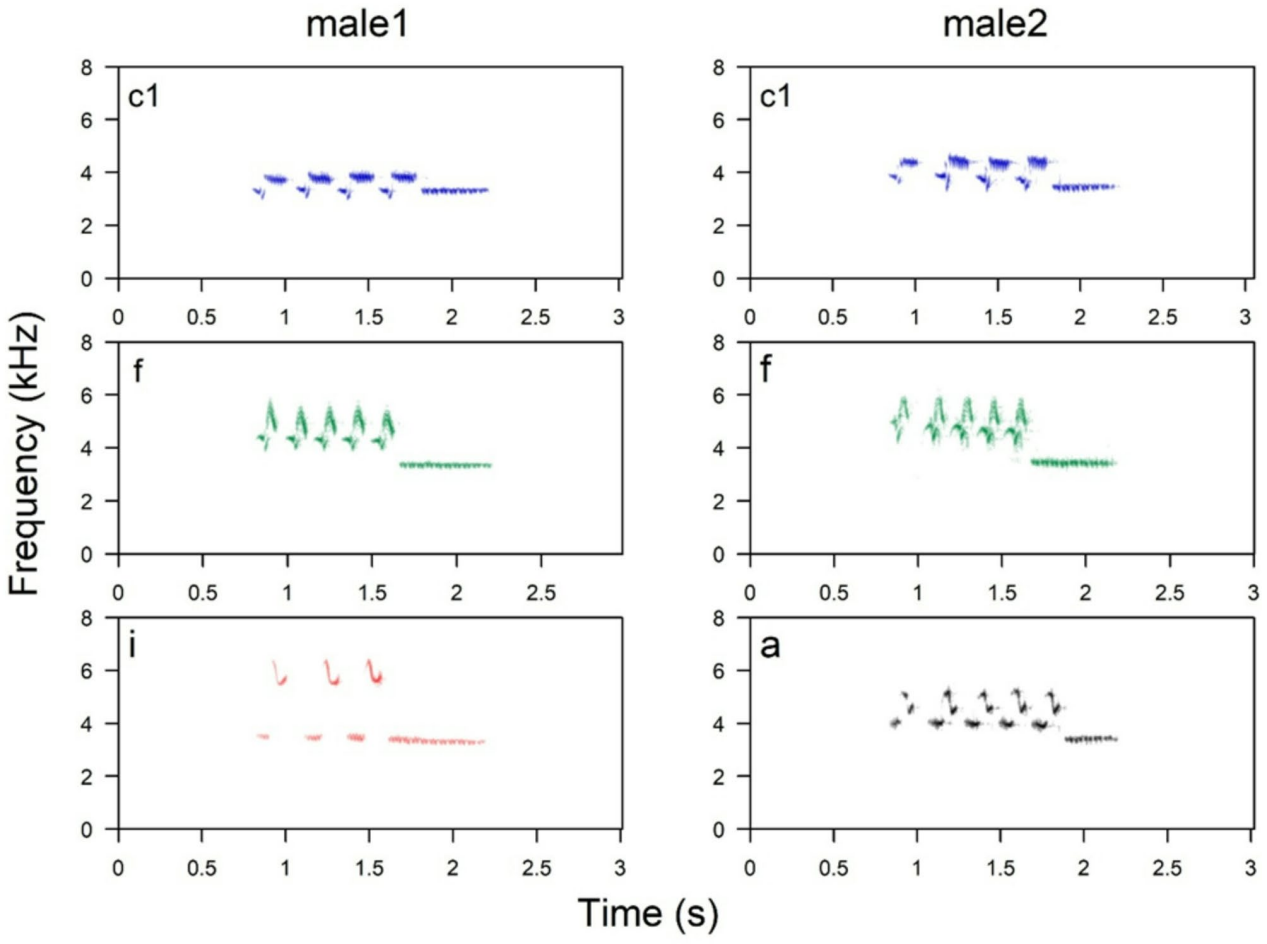
Spectrograms of three song types from the repertoires of two different males (left and right plots) recorded in the studied population. Both males share the “c1” and “f” song type.

Across Europe, Ortolan Bunting songs feature a common syntax characterised by distinct initial and final phrases, with the final phrase typically exhibiting lower frequency and a narrower bandwidth than the initial one^46^. Each phrase consists of repeated, specific syllable types that are easily distinguishable by their shapes on spectrograms during visual inspection (Fig. 1). The syllable type repeated in the IP defines a song type, and the individual’s repertoire usually includes from 1 to 4 song types. The repertoire (given song types) can be partially shared between individuals, whereas the FP is common across the entire population^46^ (Figs. 1 and 2).

### Song recordings and body size measurements

Acoustic and morphometric data were collected during three consecutive breeding seasons from 2022 to 2024. Each year, fieldwork commenced a few days after the arrival of Ortolan Buntings from their wintering grounds. This timing ensured that only males with established breeding territory boundaries were sampled. Recordings of spontaneously singing males were conducted from up to one hour before sunrise until 11:00 a.m. For each male, we aimed to record at least 50 songs to assess repertoire size reliably. A parabolic microphone Telinga Pro 7 (Telinga, Botarbo, Sweden) connected to a digital recorder, MixPre-3 I (Sound Devices, Madison, Wisconsin, USA), was utilised, and.wav files were saved at a sampling frequency of 48 kHz and a resolution of 24 bits. Geographic coordinates were recorded using GPS devices (Garmin GPSmap 60CSx and Garmin Oregon 450) for each male at the most commonly used songpost.

Each male was mist-netted using playback of an unfamiliar male’s song and marked with a unique combination of colour rings for individual identification. Basic morphological measurements were taken for each male, including wing and tail length with a metal ruler (± 0.5 mm), beak height, beak length, head-beak length, and tarsus length using callipers (± 0.1 mm), and mass using a 100 g Pesola scale (± 0.5 g). To summarise the morphological variation within the population, we calculated the mean, standard deviation (SD), and coefficient of variation for each measured trait (see Supplementary Table S1). Post-capture, males were recorded to allow for correlation of the song parameters with the body size of identified individuals.

### Acoustic analysis

In the first step, the recordings were visualized in Raven Pro 1.6 (Cornell Lab of Ornithology, K. Lisa Yang Center for Conservation Bioacoustics) using the following spectrogram parameters: window type Hamming, 1024 samples, 3dB filter bandwidth of 61 Hz; time grid 85% overlap, resulting in a hop size of 154 samples; frequency grid DFT size of 1024 samples, providing a resolution of 46.9 Hz by 10.7ms. Then, a single observer (LZ) visually inspected all the songs of each male and classified the syllable types based on their patterns in the spectrograms to determine the repertoire size (Fig. 2).

Afterwards, we extracted 9.79 ± 1.14 (mean ± SD) songs with the highest signal-to-noise ratio per song type from each male’s repertoire by separately selecting the initial and final phrases. The extracted songs were then imported in Avisoft-SASLab Pro Software v.5.02 (Avisoft Bioacoustics, Berlin, Germany) with the following settings: 1024 FFT-length, Frame [%] = 25, Window = Hamming, and Temporal Overlap = 87.5%, resulting in a bandwidth of 244 Hz, frequency resolution of 46 Hz, and time resolution of 2.67 ms^49^. Spectral characteristics were measured using the amplitude spectrum (linear) with Hamming evaluation window, a resolution of 0.366 Hz, and a peak detection threshold of −18 dB. From each part of the song spectrum, we extracted the frequency of maximal amplitude (FMA), which corresponds to the peak frequency, as well as the minimum (MINF) and maximum (MAXF) frequencies, defined as the lower and upper bounds of the frequency range exceeding the − 18 dB threshold, and the bandwidth (BAND) as the difference between MAXF and MINF. All frequency parameters (in Hz) were first log10-transformed, as frequency perception and vocal production are more accurately represented on a logarithmic scale^50^. Based on these log-transformed values, we recalculated bandwidth and derived additional traits. First, to assess the consistency of frequency parameters across repeated renditions of the same song type by the same individual, we calculated both the coefficient of variation (CV = SD/mean × 100) and repeatability estimates (intraclass correlation coefficients, ICCs) for each frequency parameter. See supplementary Tables S3 and S4 for details. After confirming individual repeatability for most of the frequency parameters, we averaged the log-transformed frequency variables across all renditions of each song type within an individual’s repertoire, obtaining a single value per acoustic parameter and song type. Next, to assess vocal performance, we calculated the sound frequency range within each male’s repertoire as the difference between the highest (MAXF) and lowest frequency (MINF) values, separately for the initial phrase (ΔIP), final phrase (ΔFP), and the overall individual repertoire (ΔF).

### Statistical analysis

All statistical analyses were conducted using R version 4.4.1 (R Core Team, 2024). We used separate Generalised Linear Models (GLMs) and Linear Mixed-Effects Models (LMMs), depending on the structure of the dataset, to examine the relationship between sound frequency characteristic of IP and FP and body size. Since the size of the body is, in essence, a multidimensional parameter, analyses that consider this characteristic can be diverse. For simplicity, we decided to include in the main text models with the most commonly used body size proxy measurement, i.e. tarsus length. To satisfy readers who prefer different approaches, we also present two analogous but slightly different analyses in the supplementary materials. Firstly, we conducted a principal component analysis (PCA) of all morphometric traits. However, due to relatively low inter-trait correlations (see Supplementary Fig. S1), a limited internal coherence (Kaiser-Meyer-Olkin [KMO] = 0.56), and the first two components with eigenvalues over 1 explained only 54% of the total variance, we decided not to use PC1 as the main body size proxy. Summary statistics for morphological traits are reported in Supplementary Table S1, and the full PCA results are provided in Supplementary Table S2. Secondly, we also calculated the Scaled Mass Index (SMI) representing body condition estimate, to assess whether condition better predicted sound frequency than body size. We calculated SMI following^51^:

$$\:SMI(\text{i)\:=\:M}i\:\times\:\:\left(\genfrac{}{}{0pt}{}{L0}{Li}\right)$$^bSMA^.

where M_i_ is the individual’s body mass, L_0_ is the mean tarsus length across the population, L_i_ is the individual’s tarsus length, and _b_SMA is the scaling exponent from an SMA regression of log body mass on log tarsus length.

Model’s results using SMI and PC1, as an alternative fixed effect respect to tarsus length are reported in the supplementary materials.

We run multiple and distinctive models with different sound frequency measurements as dependent variables according to the different song structures of the two song components. For IPs, which are frequency-modulated, we selected FMA, MINF, MAXF and BAND as dependent variables. For FPs, composed of narrowband tonal elements, we used only FMA, based on its strong significant correlation with other measures. Parameter selection was guided by pairwise correlation analyses (Figure S2) to minimise redundancy while retaining biological relevance. In the models for the initial phrase (IP), we included song type, along with tarsus length, as fixed effect due to its influence on sound frequency variation (one-way ANOVAs; R² = 0.42–0.93, all *p* < 0.00001). In contrast, for the final phrase (FP), which is shared across song types, song type explained a little portion of the total variance (one-way ANOVAs; R² < 0.18, all *p* > 0.26) and therefore was excluded from the models. The site was also included as a fixed effect in FP models to account for the spatial variation of frequency parameters. A Type III ANOVA revealed a significant effect of site on FMA (F₃,₁₁₁ = 36.28, *p* < 0.001). Year was excluded from the final models as it did not significantly explain variation in any frequency parameter across both song components (e.g., F₁,₁₁₄ = 0.03–0.97 for IP and F₁,₁₁₃ = 1.53 for FP, all *p* > 0.21).

To further explore the relationship between body size and song frequency while accounting for shared song types, we run additional models using z-scores of each raw frequency measure as dependent variables. Specifically, for each shared song type, an individual’s FMA, MAXF, MINF, and BAND values log10 (Hz), already averaged across renditions, were standardised by subtracting the population mean and dividing by the standard deviation of the corresponding song type. The resulting z-scores (FMA_zscore, MAXF_zscore, MINF_zscore, and BAND_zscore) quantified each individual’s deviation from the population mean, effectively addressing individual variation within shared song types. Since z-scores already account for song type differences, we did not include song type as a fixed effect in these models. Unlike the models using raw frequency values, which adjust for mean differences across song types via fixed effect , this approach standardises both the mean and variance, allowing a focused test of whether body size predicts individual-level variation within the same song type.

We run these models on two datasets: the “full repertoire” dataset, which included all song types sung by each male, and the “lowest-frequency song type” dataset, which retained only the song type with the lowest MINF per male, representing the individual’s minimum frequency production. For both datasets, the IP and FP were analysed separately.

For the full repertoire dataset, individual identity was included as a random factor when males had multiple song types . In contrast, it was excluded from the lowest-frequency dataset, which contained only one entry per male . Model formulas are summarised in the results section (Table 1). Bonferroni corrections were applied to sets of models sharing the same structure, dataset, and predictors to control for multiple comparisons.

**Table 1:** Summary of generalized linear and linear mixed-effects models testing the relationship between tarsus length and acoustic frequency parameters in Hz log10 transformed. Separate models were fitted for each response variable across four datasets: the initial phrase (IP) and final phrase (FP) from the full song repertoire, and the corresponding subsets restricted to the lowest-frequency song type. Tarsus length was included as a fixed effect in all models. In the full repertoire datasets, individual identity (*id*) was included as a random intercept when multiple song types were available per individual. Song type was included as a fixed effect in models relative to the IP dataset where the response variable was not a z-score. For FP models, site was also included as a fixed effect to account for significant inter-site differences in frequency parameters. Bonferroni-adjusted p-values are reported in “adjusted p-value” column only for sets of models sharing the same dataset and identical model formula, reported in the corresponding column.

FULL REPERTOIRE DATASET									
	Response variable	Estimate	Std. Error	t-value	*p*-value	adjusted *p*-value	Random effect (id) variance	Model formula	
**IP**	FMA	0.0062	0.0047	1.3220	0.1894	0.7574	0.0000	FMA ~ TARSUS + song_type + (1 | id)	
	MINF	0.0073	0.0045	1.6150	0.1127	0.4509	0.0002	MINF ~ TARSUS + song_type + (1 | id)	
	MAXF	0.0073	0.0036	2.0140	0.0503	0.2011	0.0001	MAXF ~ TARSUS + song_type + (1 | id)	
	BAND	0.0077	0.0194	0.3980	0.6903	1.0000	0.0032	BAND ~ TARSUS + song_type + (1 | id)	
	FMA_zscore	0.1620	0.1327	1.2210	0.2290	0.6869	0.0050	FMA_zscore ~ TARSUS + (1 | id)	
	MINF_zscore	0.2151	0.1536	1.4000	0.1670	0.5016	0.2348	MINF_zscore ~ TARSUS + (1 | id)	
	MAXF_zscore	0.2662	0.1451	1.8350	0.0730	0.2190	0.1372	MAXF_zscore ~ TARSUS + (1 | id)	
**FP**	FMA	0.0004	0.0018	0.2020	0.8409		0.0001	FMA ~ TARSUS + site + (1 | id)	
	FMA_zscore	−0.1016	0.1321	−0.7690	0.4460		0.2393	FMA_zscore ~ TARSUS + site + (1 | id)	
**LOWEST FREQUENCY SONG TYPE DATASET**									
**IP**	FMA	0.0077	0.0080	0.9700	0.3380	1.0000		FMA ~ TARSUS + song_type	
	MINF	0.0094	0.0056	1.6620	0.1043	0.4172		MINF ~ TARSUS + song_type	
	MAXF	0.0089	0.0045	1.9710	0.0557	0.2227		MAXF ~ TARSUS + song_type	
	BAND	0.0170	0.0145	1.1750	0.2400	1.0000		BAND ~ TARSUS + song_type	
	FMA_zscore	0.1966	0.2069	0.9500	0.3470	1.0000		FMA_zscore ~ TARSUS	
	MINF_zscore	0.2742	0.1928	1.4220	0.1610	0.4843		MINF_zscore ~ TARSUS	
	MAXF_zscore	0.4118	0.1915	2.1500	0.0366	0.1098		MAXF_zscore ~ TARSUS	
**FP**	FMA	0.0008	0.0018	0.4680	0.6420			FMA ~ TARSUS + site	
	FMA_zscore	0.0108	0.1633	0.0660	0.9480			FMA_zscore ~ TARSUS + site	

Finally, we fitted separate Gamma regression models to test whether tarsus length predicted frequency range (ΔIP, ΔFP, ΔF). Assumptions for all models, including homoscedasticity, error distribution, and absence of overdispersion or outliers, were assessed using simulated residuals with the DHARMa package (v. 0.4.6)^52^.

## Results

**Fig. 3:**
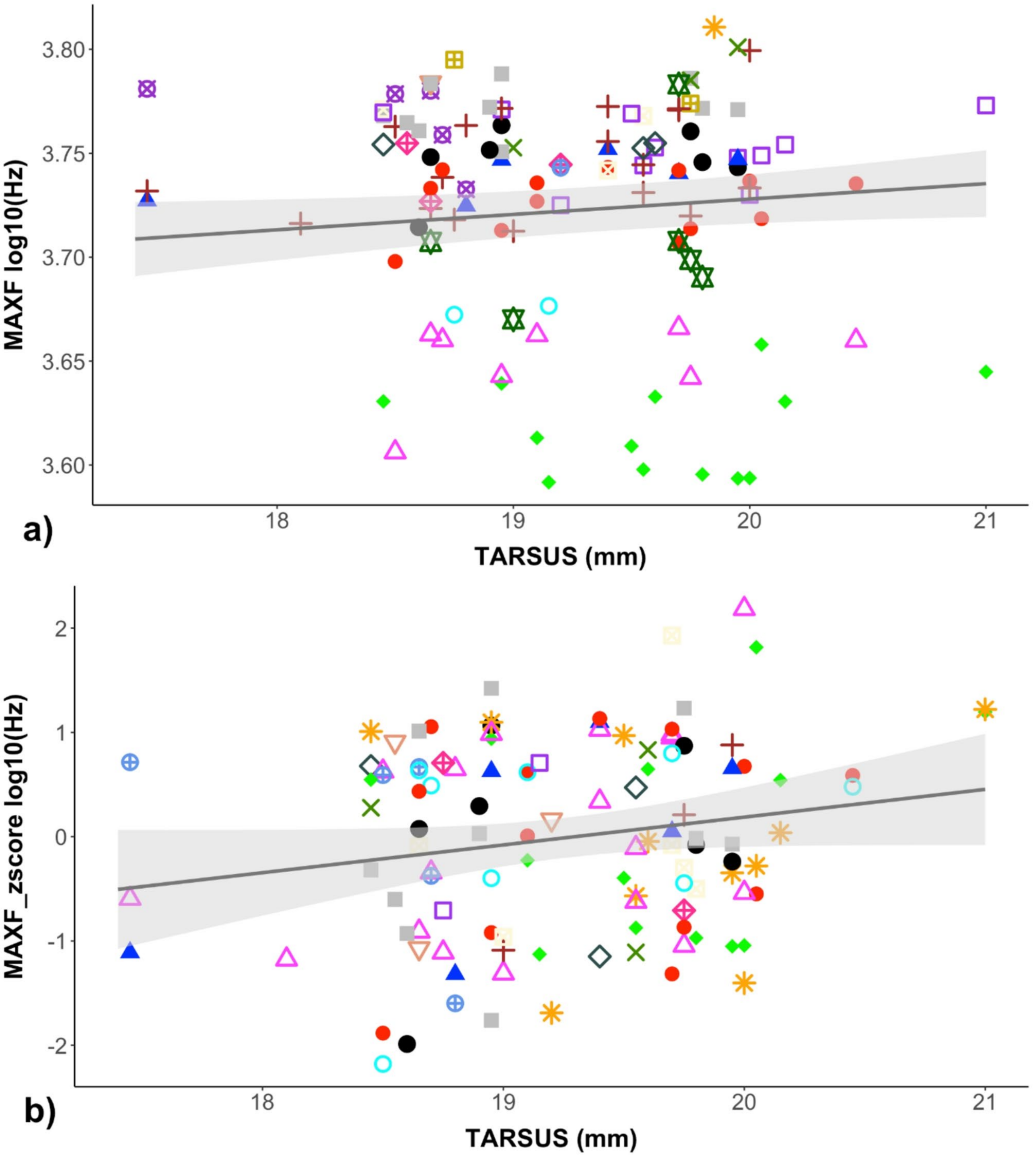
On the top **(a)** the relationship between tarsus length and the FMAX log10 transform for the initial phrase (IP). On the bottom **(b)** the relationship between tarsus length and the MAXF_zscore for the initial phrase (IP). Each data point represents the average value of a specific song type within an individual repertoire (number of individuals = 51). The same song types are distinguished by a unique combination of shape and color.

A total of 1133 songs were analysed from 51 captured males. Among these songs, 19 distinct song types could be recognised with majority of males (92%) having repertoires consisting of two or three song types (mean ± SD: 2.36 ± 0.61). Among the recorded males, 30 were measured in 2022, 16 in 2023, and 5 in 2024. Morphological traits showed limited variation within the population, with coefficients of variation generally below 6% except for beak length (CV = 11.72%) (Supplementary Table S1). Frequency traits showed high within-individual consistency and inter-individual variability, particularly in the Initial Phrase (IP), as confirmed by low intra-individual coefficients of variation (CVs) and high intraclass correlation coefficients. Results for the Final Phrase (FP) were more variable, with generally lower repeatability across traits (Supplementary Table S3 and S4). We found no significant relationship between frequency parameters and body size for either the IP or the FP analyses in the full repertoire dataset (Table 1). Both the log-transformed raw frequency parameters and the corresponding standardised (z-score) values showed no significant association with tarsus length. Although the maximum frequency in the IP dataset showed a marginal effect, including in its standardized version (MAXF: estimate = 0.0073, SE = 0.0036, t = 2.014, *p* = 0.050; MAXF_z-score: estimate = 0.2662, SE = 0.1451, t = 1.835, *p* = 0.073; Fig. 3), neither result remained significant after correction for multiple testing (Table 1). When the analysis was restricted to the lowest-frequency song type per individual, the results remained consistent (Table 1).

The regression analyses showed no significant relationship between body size and vocal performance proxies derived from frequency ranges (Table 2). Consistent with the main analyses using tarsus length, models using principal component scores (PC1) as an alternative body size index, and the Scaled Mass Index (SMI) as a proxy for body condition, also revealed no significant associations with any frequency parameter across datasets (Supplementary Tables S5–S6). All model assumptions were checked and met.

**Table 2:** Results of the gamma generalized linear model examining the relationship between Tarsus length and log10-transformed frequency range. Frequency range was calculated as the difference between the highest (MAXF) and lowest (MINF) sound frequencies within an individual repertoire, separately for the initial phrase (ΔIP), final phrase (ΔFP), and the entire song repertoire (ΔF). Models were fitted using a gamma distribution with a log link.

Response variable	Estimate	Std. Error	t value	*p* value
ΔIP	−0.0551	0.0399	−1.3820	0.1730
ΔFP	−0.1001	0.0903	−1.1080	0.2730
ΔF	−0.0531	0.0335	−1.5810	0.1210

## Discussion

We found no significant relationships between male Ortolan Buntings’ body size or body condition and the frequency characteristics of their songs. Sound frequency measures did not consistently correlate with tarsus length, our proxy for body size, nor with the Scaled Mass Index (SMI), our proxy for body condition. This is true across different song components and analyses, including those accounting for variation in song types within individuals. These findings suggest that factors beyond size may influence sound frequency in this species.

To date, studies that directly tested the relationship between song frequency and body size in species with vocal repertoire have found mixed results. Among passerines, size-frequency allometry has been revealed in Barn Swallow *Hirundo rustica*^53^Willow Warbler *Phylloscopus trochilus*^25^ and Chiffchaff *Phylloscopus collybita*^24^. However, these studies focused on specific song elements or measurements averaged across syllable types, without explicitly accounting for variation among distinct song types or elements within an individual repertoire. Hall et al.^22^accounting for vocal diversity of the common trill song types in the Purple-Crowned Fairy-Wren (*Malurus coronatus coronatus*), but still neglecting repertoire sharing, found that the lowest-frequency trill type was a reliable indicator of male body size. In contrast, Cardoso et al.^54^examining two oscine species with differing repertoire sizes, the Dark-eyed Junco (*Junco hyemalis*) and the Serin (*Serinus serinus*), and accounting for song sharing in the first species, found no correlation between body size and the sound frequency of any song or syllable type. More recently, Liu et al.^28^identified an unexpected positive relationship between tarsus length and frequency across different song types in male Dusky Warblers (*Phylloscopus fuscatus*), suggesting that the maximum frequency of entire songs could signal body size. However, in this study the variation among song types was not considered even though the male Dusky Warblers have large repertoires (up to 40 song type per male)^55^. Moreover, the association between size and frequency may be indirect, as higher maximum frequencies could arise as a byproduct of producing rapid, wide-bandwidth trills, which are condition-dependent signals^56^. In our study, maximum frequency similarly showed a marginal positive association with tarsus length in the initial phrase, but this relationship did not remain significant after correction for multiple comparisons, suggesting it may reflect a weak or incidental trend rather than a robust signal of male size. By explicitly considering song type sharing and variation, our study advances this debate, providing a more comprehensive, though still limited, understanding of whether body size and condition might affect vocal traits in species with small repertoires.

The absence of a relationship between body size or body condition and song frequency may result from several factors. It is possible that limited variation in body size within our population constrained our ability to detect size-related effects^27,54^. Alternatively, song-learning processes and other selective pressures may override morphological constraints on frequency production. As suggested for many oscine species^13^the complex control of vocal production afforded by the syrinx, combined with vocal learning, can decouple sound frequency from physical body traits. In many song-learning species, the first level of sound frequency selection likely arises during song acquisition, when young males learn by copying the songs of their fathers or neighbouring males^3,42,57^. Moreover, in the Ortolan Bunting, evidence of mimicry of other species has been documented, supporting the occurrence of song flexibility in this species^58^. Variations in frequency may result from inaccuracies during the copying process, anatomical differences, or other pressures such as selection for vocal identity^59^. Since body size does not seem to constrain sound frequency in this species, frequency differences, particularly in shared song types, might reflect the need for males to stand out from a competitive acoustic space^60^. Once the repertoire is fixed by the learning process, selection may favour frequency variation that enhances individual recognition. The “dear enemy effect”, where territorial neighbours reduce aggression once recognition is established, emphasises the importance of distinct vocal signatures in reducing costly conflicts^61,62^. Previous studies on Ortolan Buntings, as well as our data (see Supplementary Tables S3–S4 for repeatability results), revealed that even in shared song types, both the initial and final phrases, retain individual distinctiveness, particularly through frequency parameters^45^.

While our findings suggest that factors beyond body size influence song frequency, they do not exclude its potential role in signalling other aspects of individual quality. The distinct acoustic properties of the initial and final phrases may reflect functional differences. Site-specific variation in FP frequency likely reflects the song-learning process, where young males imitate local tutors. This could make the FP frequency informative about population origin, aligning with the “song sharing hypothesis”^59^which proposes that females prefer locally adapted males, more familiar with available resources, and likely to hold established territories^63^. In our study population, across-year observations confirm that ringed males consistently return to the same capture site. Maintaining consistent FP frequency could therefore benefit males, potentially explaining the lack of a size-related trend. In contrast, the initial phrase, which defines individual repertoires, may be more flexible and subject to different and non-exclusive selective forces, like mate attraction and territory defence^64^. Even without a direct link to body size, frequency can still convey information about individual quality. Higher sound frequency songs are more challenging to sustain at high amplitude due to biomechanical constraints, such as increased sub-syringeal air pressure and greater demands on vocal control, making them a potential indicator of motor performance or condition^29,32,65^. Conversely, lower sound frequency vocalisations are widely associated with aggression and dominance in birds^13^ and other taxa^66,67^. Younger and smaller individuals typically produce higher-pitched signals^68^reinforcing the perception that lower frequencies indicate maturity and higher status. This pattern also extends to human speech, where lower formant dispersion is perceived as more attractive, authoritative, and dominant^69,70^.

The absence of a relationship between body size and song frequency in our data does not allow us to conclude whether body size reflects individual quality. While one possibility is that, in Ortolan Buntings as in other song-learning species, individual quality is expressed through song traits other than frequency^7^it is also possible that larger body size does not offer a selective advantage in this species. Indeed, in some species, smaller-sized males benefit from greater agility, particularly in courtship displays or other behaviours that are relevant for sexual selection^71,72^. Clarifying whether frequency, or other song traits, acts as an honest signal of quality will require linking acoustic variation to fitness metrics such as mating success or offspring viability.

This study challenges the assumption that larger males consistently produce lower sound frequencies vocalisations, traditionally viewed as reliable indicators of male body size, and shows no evidence of a frequency-size link in this song-learning species.

Future research should focus on larger and more diverse populations and consider factors such as song type diversity and sharing patterns, learning histories, and population density. Targeted empirical studies are necessary to identify the selective pressures shaping frequency variation and its role in communication. Overall, this study provides new evidence to the relatively underexplored role of body constraints in song-learning species and offers a framework for better disentangling the signalling role of sound frequency.

## Electronic supplementary material

Below is the link to the electronic supplementary material.

Supplementary Material 1

Supplementary Material 2

Supplementary Material 3

Supplementary Material 4

Supplementary Material 5

Supplementary Material 6

Supplementary Material 7

## Data Availability

Dataset analysed and the corresponded R script are available in the supplementary materials.
