# Supplementary material for: Territorial song frequency does not signal body size in a song-learning passerine
Source: Sci Rep. 2025 Jul 16;15:25774. doi: 10.1038/s41598-025-11589-4 (PMC12267584; doi:10.1038/s41598-025-11589-4)
Supplement: Supplementary file 4 — Supplementary Material 4 [file 41598_2025_11589_MOESM4_ESM.docx]

**Information about supplementary material**

**df_morpho_body_size.csv :**

This dataset contains morphological measurements and body size estimates for 51 individual Ortolan Buntings, recorded across multiple years and sites. It includes 15 variables, with key measurements such as wing, tail, tarsus, beak height, beak length, beak head, and weight. Body size condition is represented by the Size-Mass Index (SMI), calculated following Peig & Green (2009), and the first principal component (PC1), derived from a PCA on all morphological traits. Each observation is identified by a unique “id” and includes the year, site, date, and time of the measurement.

**EH_22_23_24_raw_acoust_data.csv :**

This dataset contains raw acoustic measurements of 1133 songs extracted from 51 captured males. For each male (identified by "id"), there are 9.79 ± 1.14 (mean ± SD) songs per song type ( classified by “song_type” column), with the initial (IP) and final (FP) phrases separated in the "df" column. Each song is also associated with a specific song number ("song_num"). Key acoustic parameters such as the frequency of maximal amplitude (FMA), minimum frequency (MINF), maximum frequency (MAXF), spectral centroid, bandwidth, and quartile frequencies (q25, q50, q75) were extracted from the amplitude spectrum using Avisoft-SASLab Pro software. Additionally, the dataset includes the total repertoire size for each male.

**EH_22_23_24_data_all_song.csv:**

This dataset contains processed data derived from the previous raw acoustic measurements. For each male (identified by "id"), we averaged all renditions of each song type within their repertoire, resulting in a single value per acoustic parameter for each song type. Frequency parameters include both original and log-transformed versions (denoted by “_log”). Additionally, frequency range parameters are included:

- **deltaF**: The difference between the highest (MAXF) and lowest (MINF) frequencies, separately for the initial (ΔIP) and final (ΔFP) phrases, depending on the "df" entry.
- **deltaF_ID**: The difference between the highest (MAXF) and lowest (MINF) frequencies across all song types of an individual, independently of the phrase, referred to as ΔF in the manuscript.

To account for individual variation within shared song types, we standardized the FMA, MAXF, MINF, and BAND values by subtracting the population mean and dividing by the standard deviation. The resulting z-scores (FMA_zscore, MAXF_zscore, MINF_zscore, and BAND_zscore) reflect each individual’s deviation from the population mean.

This dataset contains the morphological traits and body size estimates for each individual, corresponding to those included in the dataset “df_morpho_body_size.csv”.

**EH_22_23_24_data_lowest_song.csv**

This dataset is a filtered version of the previous one, containing acoustic measurements for the lowest-frequency song type produced by each male, specifically the song type with the lowest MINF.

**EH_freq_bodysize_SciRep_complete.R**

This file contains the complete R script used to process and analyze the acoustic and body size data, from the raw measurements (“df_morpho_body_size.csv” and “EH_22_23_24_raw_acoust_data.csv”) to the final dataset used in the statistical models. The section specifically dedicated to model fitting and testing is located from line 586 to line 1317. In this part of the script, the names of the code sections correspond to those used in Table 1 of the results section, where the model outputs are reported.
