# Supplementary material for: Territorial song frequency does not signal body size in a song-learning passerine
Source: Sci Rep. 2025 Jul 16;15:25774. doi: 10.1038/s41598-025-11589-4 (PMC12267584; doi:10.1038/s41598-025-11589-4)
Supplement: Supplementary file 7 — Supplementary Material 7 [file 41598_2025_11589_MOESM7_ESM.docx]

| **Traits** | **N** | **Mean** | **SD** | **CV (%)** |
| --- | --- | --- | --- | --- |
| Wing (mm) | 51 | 89.33 | 2.49 | 2.78 |
| Tail (mm) | 51 | 67.93 | 2.02 | 2.98 |
| Tarsus (mm) | 51 | 19.29 | 0.66 | 3.45 |
| Beak height (mm) | 51 | 5.32 | 0.28 | 5.26 |
| Beak lenght (mm) | 51 | 11.19 | 1.31 | 11.72 |
| Beak head (mm) | 51 | 29.86 | 0.67 | 2.24 |
| Weight (g) | 47 | 22.35 | 1.32 | 5.93 |

**Supplementary Table S1.** Summary statistics for morphological traits, including sample size (N), mean, standard deviation (SD), and coefficient of variation (CV = SD / Mean × 100) for each trait.

| **Variables** | **PC1  loading** | **PC1  contribution (%)** | **PC2 loading** | **PC2  contribution (%)** | **MSA (KMO)** |
| --- | --- | --- | --- | --- | --- |
| Wing (mm) | 0.702 | 21.18 | 0.601 | 24.63 | 0.53 |
| Tail (mm) | 0.534 | 12.27 | 0.764 | 39.87 | 0.47 |
| Tarsus (mm) | 0.658 | 18.61 | -0.435 | 12.94 | 0.62 |
| Beak height (mm) | 0.711 | 21.73 | -0.175 | 2.09 | 0.70 |
| Beak length (mm) | -0.049 | 0.10 | 0.123 | 1.03 | 0.30 |
| Beak head (mm) | 0.552 | 13.1 | -0.42 | 12.07 | 0.55 |
| Weight (g) | 0.55 | 13.02 | -0.329 | 7.38 | 0.60 |
| Eigenvalue |  | 2.33 |  | 1.47 |  |
| Variability (%) |  | 33.25 |  | 20.93 |  |
| Cumulative (%) |  | 33.25 |  | 54.17 |  |
| Overall KMO MSA |  |  |  |  | **0.56** |

**Supplementary Table S2.** Loadings (coordinates) and percentage contributions of each morphological variable to the first two principal components (PC1 and PC2), followed by eigenvalues, the percentage of explained variance, and cumulative variance. The Kaiser-Meyer-Olkin (KMO) measure of sampling adequacy is also reported for each morphological variable, along with the overall value.


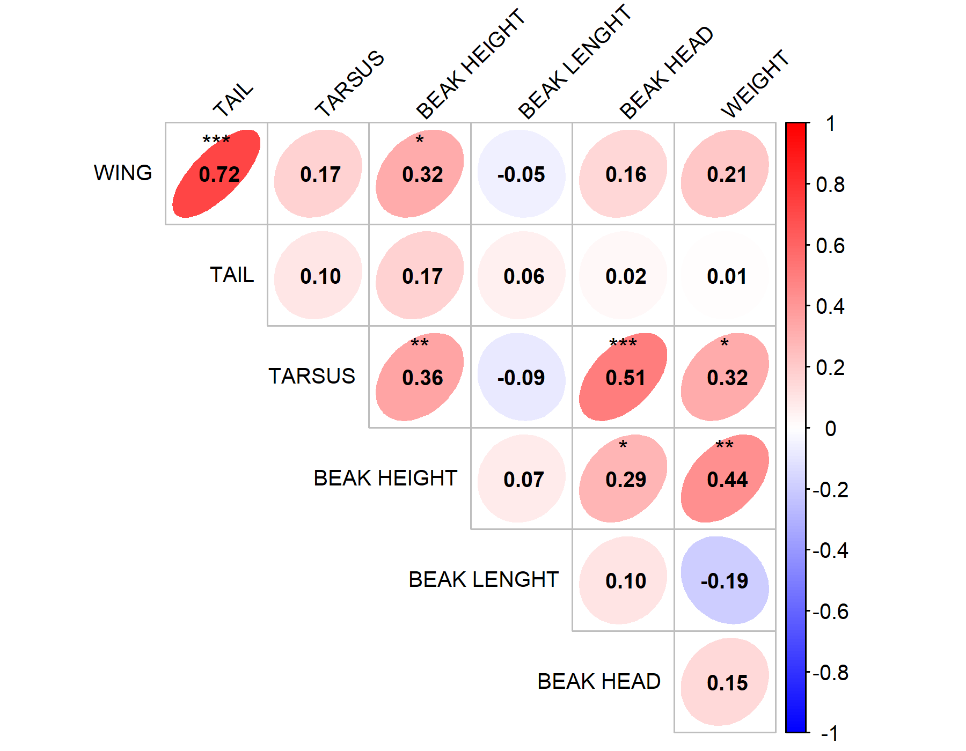


**Supplementary Fig. S1.** Pairwise Pearson correlations among morphological traits. The plot displays correlation coefficients in each cell using ellipses to indicate the strength and direction of the correlation (red: positive, blue: negative). Asterisks above the coefficients indicate the significance level of each correlation: (* p < 0.05; ** p < 0.01; *** p < 0.001).

|  | **IP** | | **FP** | |
| --- | --- | --- | --- | --- |
| **Traits** | **Intra-CV** | **Inter-CV** | **Intra-CV** | **Inter-CV** |
| Bandwidth (Hz) | 6.81 | 36.64 | 24.36 | 42.72 |
| FMA (Hz) | 4.81 | 8.22 | 0.68 | 2.59 |
| MAXF (Hz) | 1.50 | 11.54 | 1.52 | 3.52 |
| MINF (Hz) | 1.71 | 13.12 | 0.89 | 2.66 |

**Supplementary Table S3.** Coefficient of variation (CV = SD/mean × 100) calculated with raw frequency values for the Initial phrase (IP) and final phrase (FP). Intra-CV represent the mean of intra-individual CVs, calculated across repeated renditions for each individual:song type combination (9.79 ± 1.14 renditions per unit; mean ± SD). Inter-CV indicates the inter-individual CV, calculated across individuals using the mean trait value per individual:song type combination.

|  | ICC | |
| --- | --- | --- |
| **Trait** | **IP** | **FP** |
| FMA log10 (Hz) | 0.51 | 0.83 |
| MINF log10 (Hz) | 0.92 | 0.67 |
| MAXF log10 (Hz) | 0.95 | 0.45 |
| Bandwidth log10 (Hz) | 0.91 | 0.13 |

**Supplementary Table S4.** Repeatability estimates for log10 transformed frequency traits in the Initial phrase (IP) and final phrase (FP). Intraclass correlation coefficients (ICCs) were calculated using linear mixed-effects models with individual:song type combination as a random factor (9.79 ± 1.14 renditions per unit; mean ± SD) and each trait as a dependent variable. ICC values quantify the proportion of variance attributable to consistent differences among individuals across repeated renditions. Higher values indicate greater within-individual consistency. The analysis followed the framework described by Nakagawa & Schielzeth (2010) *Biological Reviews*, *85*(4), 935-956.


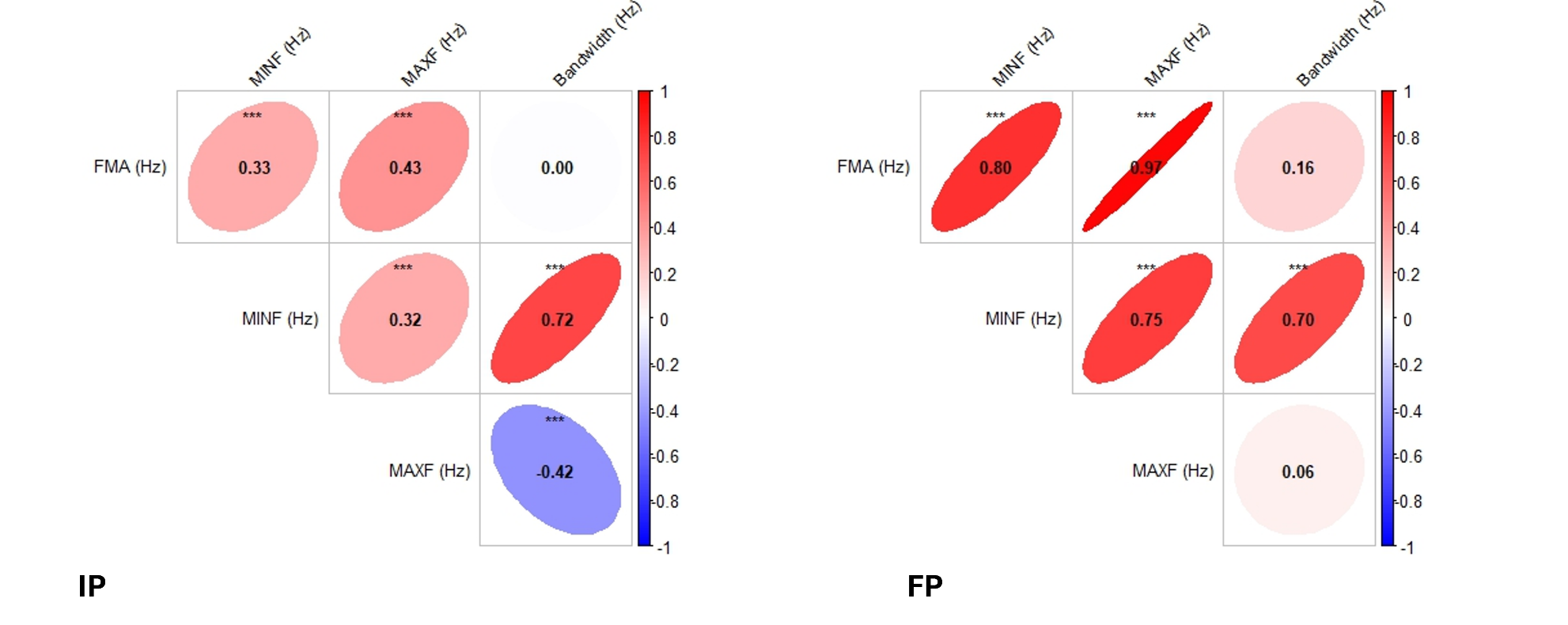


**Supplementary Fig. S2.** Pearson correlation matrices for acoustic frequency parameters in Hz measured in the Initial phrase (IP) and Final phrase FP (FP). Ellipses indicate the strength and direction of correlations (red: positive, blue: negative). Numerical values represent correlation coefficients, and asterisks denote significance levels (*p* < 0.05: *, *p* < 0.01: **, *p* < 0.001: ***)

| **FULL REPERTOIRE DATASET** | | | | | | | | |  |
| --- | --- | --- | --- | --- | --- | --- | --- | --- | --- |
|  |  |  |  |  |  |  |  |  |  |
|  | **Response variable** | **Estimate** | **Std. Error** | **t-value** | **p-value** | **adjusted  p-value** | **Random effect  (id) variance** | **Model formula** |  |
| **IP** | FMA | -0.0014 | 0.0021 | -0.6540 | 0.5149 | 1.0000 | 0.0000 | FMA ~ SMI + song_type + (1 \| id) |  |
|  | MINF | -0.0026 | 0.0021 | -1.2160 | 0.2306 | 0.9226 | 0.0002 | MINF ~ SMI + song_type + (1 \| id) |  |
|  | MAXF | -0.0010 | 0.0017 | -0.6000 | 0.5523 | 1.0000 | 0.0001 | MAXF ~ SMI + song_type + (1 \| id) |  |
|  | BAND | 0.0071 | 0.0088 | 0.8050 | 0.4207 | 1.0000 | 0.0036 | BAND ~ SMI + song_type + (1 \| id) |  |
|  | FMA_zscore | -0.0392 | 0.0619 | -0.6330 | 0.5300 | 1.0000 | 0.0125 | FMA_zscore ~ SMI + (1 \| id) |  |
|  | MINF_zscore | -0.0434 | 0.0702 | -0.6180 | 0.5390 | 1.0000 | 0.2375 | MINF_zscore ~ SMI + (1 \| id) |  |
|  | MAXF_zscore | -0.0048 | 0.0678 | -0.0710 | 0.9440 | 1.0000 | 0.1554 | MAXF_zscore ~ SMI + (1 \| id) |  |
| **FP** | FMA | 0.0005 | 0.0009 | 0.5290 | 0.6000 |  | 0.0001 | FMA ~ SMI + site + (1 \| id) |  |
|  | FMA_zscore | 0.0323 | 0.0641 | 0.5040 | 0.6170 |  | 0.2900 | FMA_zscore ~ SMI + site + (1 \| id) |  |
| **LOWEST FREQUENCY SONG TYPE DATASET** | | | | | | | | |  |
|  |  |  |  |  |  |  |  |  |  |
| **IP** | FMA | -0.0020 | 0.0035 | -0.5790 | 0.5660 | 1.0000 |  | FMA ~ SMI + song_type |  |
|  | MINF | -0.0048 | 0.0024 | -2.0020 | 0.0529 | 0.2115 |  | MINF ~ SMI + song_type |  |
|  | MAXF | -0.0040 | 0.0020 | -2.0370 | 0.0491 | 0.1963 |  | MAXF ~ SMI + song_type |  |
|  | BAND | -0.0021 | 0.0063 | -0.3270 | 0.7438 | 1.0000 |  | BAND ~ SMI + song_type |  |
|  | FMA_zscore | -0.0868 | 0.0936 | -0.9280 | 0.3580 | 1.0000 |  | FMA_zscore ~ SMI |  |
|  | MINF_zscore | -0.1339 | 0.0861 | -1.5550 | 0.1270 | 0.3811 |  | MINF_zscore ~ SMI |  |
|  | MAXF_zscore | -0.1629 | 0.0876 | -1.8600 | 0.0696 | 0.2089 |  | MAXF_zscore ~ SMI |  |
| **FP** | FMA | 0.0004 | 0.0008 | 0.4960 | 0.6220 |  |  | FMA ~ SMI +site |  |
|  | FMA_zscore | 0.0173 | 0.0777 | 0.2230 | 0.8250 |  |  | FMA_zscore ~ SMI + site |  |

**Supplementary Table S5.** Summary of generalised linear and generalised linear mixed-effects models testing the relationship between Scaled Mass Index (SMI) and acoustic frequency parameters in Hz, log10 transformed. Separate models were fitted for each response variable across four datasets: the Initial Phrase (IP) and Final Phrase (FP) from the full song repertoire, and the corresponding subsets restricted to the lowest-frequency song type. SMI was included as a fixed effect in all models. In the full repertoire datasets, individual identity (*id*) was included as a random intercept when multiple song types were available per individual. Song type was included as a fixed effect in all models where the response variable was not a z-score. For FP models, site was also included as a fixed effect to account for significant inter-site differences in frequency parameters. Bonferroni-adjusted p-values are reported in “adjusted p-value” column only for sets of models sharing the same dataset and identical model formula, reported in the corresponding column.

| **FULL REPERTOIRE DATASET** | | | | | | | | |  |
| --- | --- | --- | --- | --- | --- | --- | --- | --- | --- |
|  |  |  |  |  |  |  |  |  |  |
|  | **Response variable** | **Estimate** | **Std. Error** | **t-value** | **p-value** | **adjusted  p-value** | **Random effect  (id) variance** | **Model formula** |  |
| **IP** | FMA | 0.0006 | 0.0020 | 0.2800 | 0.7800 | 1.0000 | 0.0000 | FMA ~ PC1 + song_type + (1 \| id) |  |
|  | MINF | 0.0019 | 0.0019 | 0.9520 | 0.3457 | 1.0000 | 0.0002 | MINF ~ PC1 + song_type + (1 \| id) |  |
|  | MAXF | 0.0014 | 0.0016 | 0.8500 | 0.4002 | 1.0000 | 0.0001 | MAXF ~ PC1 + song_type + (1 \| id) |  |
|  | BAND | -0.0023 | 0.0083 | -0.2700 | 0.7865 | 1.0000 | 0.0033 | BAND ~ PC1 + song_type + (1 \| id) |  |
|  | FMA_zscore | -0.0025 | 0.0580 | -0.0430 | 0.9660 | 1.0000 | 0.0171 | FMA_zscore ~ PC1 + (1 \| id) |  |
|  | MINF_zscore | 0.0534 | 0.0666 | 0.8010 | 0.4270 | 1.0000 | 0.2409 | MINF_zscore ~ PC1 + (1 \| id) |  |
|  | MAXF_zscore | 0.0626 | 0.0636 | 0.9840 | 0.3300 | 0.9907 | 0.1532 | MAXF_zscore ~ PC1 + (1 \| id) |  |
| **FP** | FMA | 0.0001 | 0.0008 | 0.0820 | 0.9353 |  | 0.0001 | FMA ~ PC1 + site + (1 \| id) |  |
|  | FMA_zscore | -0.0411 | 0.0583 | -0.7060 | 0.4844 |  | 0.2388 | FMA_zscore ~ PC1 + site + (1 \| id) |  |
| **LOWEST FREQUENCY SONG TYPE DATASET** | | | | | | | | |  |
|  |  |  |  |  |  |  |  |  |  |
| **IP** | FMA | 0.0005 | 0.0035 | 0.1510 | 0.8810 | 1.0000 |  | FMA ~ PC1 + song_type |  |
|  | MINF | -0.0007 | 0.0025 | -0.2810 | 0.7801 | 1.0000 |  | MINF ~ PC1 + song_type |  |
|  | MAXF | -0.0003 | 0.0021 | -0.1350 | 0.8934 | 1.0000 |  | MAXF ~ PC1 + song_type |  |
|  | BAND | 0.0043 | 0.0064 | 0.6700 | 0.5031 | 1.0000 |  | BAND ~ PC1 + song_type |  |
|  | FMA_zscore | -0.0194 | 0.0904 | -0.2140 | 0.8310 | 1.0000 |  | FMA_zscore ~ PC1 |  |
|  | MINF_zscore | -0.0283 | 0.0852 | -0.3320 | 0.7410 | 1.0000 |  | MINF_zscore ~ PC1 |  |
|  | MAXF_zscore | -0.0019 | 0.0869 | -0.0210 | 0.9830 | 1.0000 |  | MAXF_zscore ~ PC1 |  |
| **FP** | FMA | 0.0002 | 0.0008 | 0.2420 | 0.8095 | 1.0000 |  | FMA ~ PC1 + site |  |
|  | FMA_zscore | -0.0010 | 0.0718 | -0.0150 | 0.9884 | 1.0000 |  | FMA_zscore ~ PC1 + site |  |

**Supplementary Table S6.** Summary of generalized linear and linear mixed-effects models testing the relationship between PC1 of morphological traits and acoustic frequency parameters in Hz log10 transformed. Separate models were fitted for each response variable across four datasets: the Initial Phrase (IP) and Final Phrase (FP) from the full song repertoire, and the corresponding subsets restricted to the lowest-frequency song type. PC1 was included as a fixed effect in all models. In the full repertoire datasets, individual identity (*id*) was included as a random intercept when multiple song types were available per individual. Song type was included as a fixed effect in all models where the response variable was not a z-score. For FP models, the site was also included as a fixed effect to account for significant inter-site differences in frequency parameters. Bonferroni-adjusted p-values are reported in the “adjusted p-value” column only for sets of models sharing the same dataset and identical model formula, reported in the corresponding column.
